# Identification of a novel *GPR143* deletion in a Chinese family with X-linked congenital nystagmus

**Published:** 2008-05-30

**Authors:** Pingtong Zhou, Zhiqiang Wang, Jing Zhang, Landian Hu, Xiangyin Kong

**Affiliations:** Institute of Health Sciences, Shanghai Institutes for Biological Sciences (SIBS), Chinese Academy of Sciences (CAS) & Shanghai Jiao Tong University School of Medicine (SJTUSM), Shanghai, China

## Abstract

**Purpose:**

To map and identify the genetic mutation underlying X-linked congenital nystagmus in a Chinese family.

**Methods:**

Genomic DNA was prepared from peripheral blood, and linkage analysis was performed using short tandem repeat (STR) polymorphism markers. We used Cyrillic software to manage pedigree and haplotype data and used MLINK to calculate LOD scores. Dye-terminator cycle-sequencing was used to detect the sequence variation of polymerase chain reaction (PCR)-amplified exons.

**Results:**

Linkage analysis mapped the disease-causing gene to Xp22.3 with a significant two-point LOD score (Z) at marker DXS7103 (Z=3.16, recombination fraction [θ]=0). Haplotype analysis in this region supported the result. In analyzing the candidate gene in the linked region, we found a 37-bp deletion in exon 1 of *GPR143* in all male patients.

**Conclusions:**

The revealed 37-bp deletion in *GPR143* is frameshift and is predicted to result in a truncated protein of 93 residues. These results indicate that this novel *GPR143* mutation is associated with the congenital nystagmus observed in this Chinese family.

## Introduction

Nystagmus is an ocular disorder characterized by repetitive and involuntary oscillation of the eyeball [1]. Visual function can be significantly reduced owing to constant eye movement, but the degree of visual impairment varies [2]. This disease can be secondary to other visual or neurological disease or can occur as an isolated inherited trait termed congenital nystagmus (CN). The condition is present at birth or develops within the first few months of life with an estimated annual incidence of 1/20,000 [3]. CN is genetically heterogeneous, and several patterns of inheritance of CN have been described including autosomal dominant [4-6], autosomal recessive [7], X-linked dominant [7], and X-linked recessive patterns [8,9]. It has been suggested that X-linked patterns of genetic inheritance with incomplete penetrance are probably most common. Three different genetic loci for X-linked CN have been mapped to chromosomes Xp22 [10], Xp11.3–11.4 [8], and Xq26-X27 [7,9].

To date, two genes, the *FERM domain-containing 7* (*FRMD7*) gene and the *G protein-coupled receptor 143* (*GPR143*) gene, have been identified as disease-causing genes for CN. *FRMD7* is at the known locus, Xq26–27, and multiple mutations of *FRMD7* have been reported since it was first identified in 2006 [11-14]. Another gene, *GPR143*, also known as the *OA1* gene at Xp22, causes ocular albinism upon mutation [15,16]. Ocular albinism is an X-linked type of albinism that mainly effects pigment production in the eye, resulting in hypopigmentation of the retina, foveal hypoplasia, reduction of visual acuity, nystagmus, and optic misrouting [15,16]. However, a recent study of a Chinese family with X-linked CN, occurring without the classical phenotype (retinal hypopigmentation) of ocular albinism but only with nystagmus (some patients also suffer from foveal hypoplasia and reduction of visual acuity), has identified a *GPR143* gene mutation [10]. It is suggested that the *GPR143* mutation may also produce CN as the most prominent manifestation.

In this study, we collected data from a four-generation Chinese family with X-linked congenital nystagmus. All the affected individuals suffer from nystagmus and amblyopia but without any sign of retinal hypopigmentation. We mapped the disease-causing gene to Xp22.3 and found a 37-bp deletion in exon 1 of *GPR143* in all affected male and female carriers. Our results indicate that this novel *GPR143* mutation might cause the X-linked CN in this family.

## Methods

### Family data and extraction of human genomic DNA

**Figure 1:**
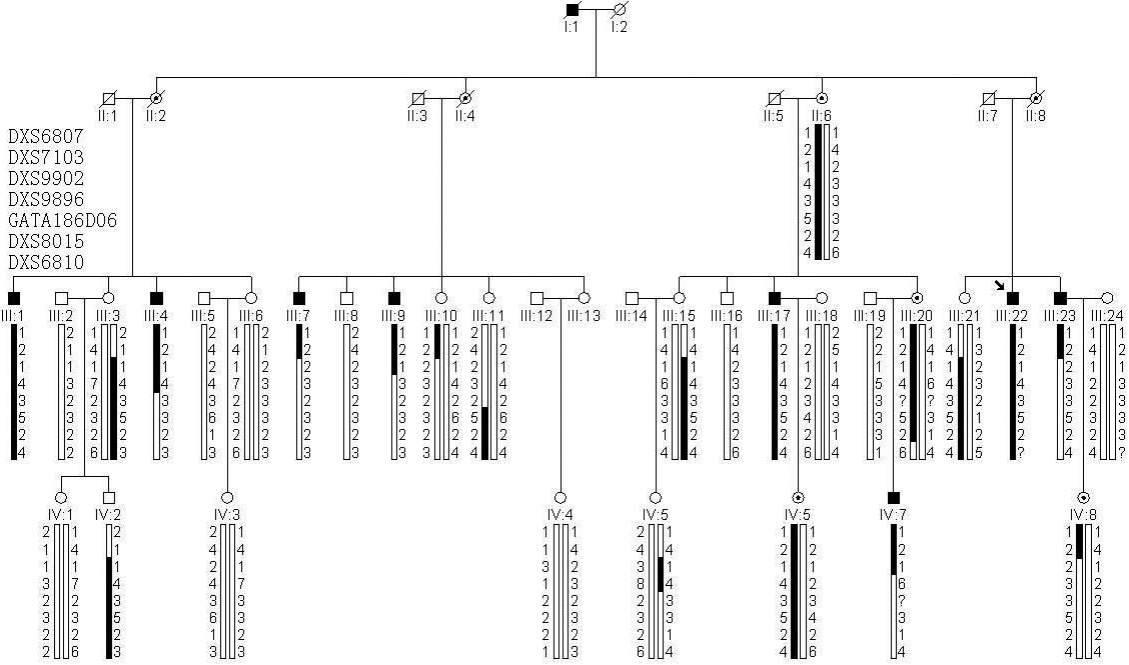
The pedigree and haplotype analysis of the Chinese congenital nystagmus subject family. The proband is marked with an arrow. Eight markers are listed from top to bottom: telomere - DXS6807 - DXS7103 - DXS9902 - DXS9896 - GATA186D06 - DXS8015 - DXS6810 - DXS8035 - centromere. Blackened regions of haplotypes indicate linkage between the markers and the disease. Question marks indicate that the genotype is not determined.

The study had the approval of the local and regional ethics committee and conformed to the tenets of the Declaration of Helsinki. A four-generation Han Chinese family with X-linked CN was identified in Zhengzhou, Henan Province, China. It consisted of 33 living members and involved nine affected males (Figure 1). All members of this family were diagnosed carefully by ocular examinations, which were performed with slit lamp biomicroscopy and direct and indirect ophthalmoscopy. One hundred unrelated Han Chinese individuals were recruited as the control subjects and were clinically examined by the Ruijing Hospital, Shanghai Jiao Tong University School of Medicine, Shanghai, China. All the individuals in the control group were healthy and without any history of familial inherited disease. Peripheral blood was obtained from 30 family members (except III:12, III:13, and III:14) and the control subjects after obtaining informed consent. The genomic DNA was prepared using the QIAmp DNA Blood Kit (Qiagen, Hilden, Germany) according to the manufacturer’s instructions.

### Genotyping and linkage analysis

Genotyping was performed using 26 fluorescent microsatellite markers covering the entire X chromosome. All of the short tandem repeat (STR) markers selected from the combined Genethon, Marshfield, and deCODE genetic linkage maps (National Center for Biotechnology Information, Bethesda, MD, NCBI) were amplified with M13-tailed primers in the presence of an IRDye800 labeled M13-universal primer (Li-Cor, Lincoln, NE) and detected with a Li-Cor 4200L DNA sequencer (Li-Cor). A two-point linkage analysis was conducted using a LINKAGE (version 5.1) software package [17]. This disease was specified as an X-linked recessive trait with penetrance of 1.0 in males, and the affected allele frequency was assumed to be 0.001. Pedigree drawing and haplotype construction were done using Cyrillic (version 2.0) software (Cyrillic software, Oxfordshire, UK).

### Mutation detection

DNA fragments encompassing the coding and adjacent intron regions of the *G protein-coupled receptor 143* (*GPR143*, OMIM 300500) gene were amplified by polymerase chain reaction (PCR) using nine pairs of gene-specific primers. Each fragment was sequenced using the ABI BigDye Terminator Cycle Sequencing kit (Applied Biosystems, Foster City, CA) and an ABI 3100 sequencer according to the manufacturer’s protocol.

## Results

### Pedigree of the X-linked congenital nystagmus family

In this family, all affected individuals were male and all obligate carriers had no sign of nystagmus (Figure 1). The disease was transmitted from female carrier to affected son and was an apparent X-linked recessive trait. We then determined the disease to exhibit an X-linked recessive inheritance pattern using linkage analysis. DNA was available from 30 family members in three generations (including nine affected individuals), and genotyping was conducted on the X chromosome.

### Linkage analysis

Upon analysis of the X chromosome scan, the highest two-point LOD score (Z) was found for marker DXS7103 (Z=3.16, θ=0). One STR marker, DXS6807, which was near DXS7103, also gave a positive LOD score of 1.28 at θ=0 (Table 1). Other markers showed LOD scores of -∞ or <1. Therefore, we mapped the critical region for CN to a 22 cM interval between the telomeric boundary and marker DXS9902. Haplotypes in this region supported the linkage results (Figure 1). Both the affected individuals, III: 7 and III: 23, had recombinations between DXS7103 and DXS9902 while the non-affected male, IV: 2, also had a recombination in this region.

**Table 1:** Two-point LOD score result between the disease gene and eight markers of chromosome X.

**Marker**	**CM**	**Two-point LOD score at θ=**						
		**0**	**0.01**	**0.05**	**0.1**	**0.2**	**0.3**	**0.4**
DXS6807	4.39	1.28	1.26	1.17	1.06	0.82	0.57	0.30
DXS7103	10.93	3.16	3.11	2.92	2.65	2.08	1.41	0.65
DXS9902	22.04	-∞	1.50	-0.23	0.21	0.44	0.37	0.15
DXS9896	39.52	-∞	-6.79	-3.43	-2.10	-0.97	-0.48	-0.23
GATA186D06	44.96	-∞	-2.95	-1.58	-1.01	-0.49	-0.23	-0.08
DXS8015	53.71	-∞	-6.57	-3.21	-1.87	-0.72	-0.23	-0.04
DXS6810	63.59	-∞	-1.64	-0.95	-0.65	-0.37	-0.20	-0.09
DXS8035	65.79	-∞	-6.89	-3.50	-2.13	-0.92	-0.36	-0.08

Z values for eight markers on chromosome Xp are shown, and the maximum Z value occurs at marker DXS7103.

### Mutation of the *GPR143* gene

**Figure 2:**
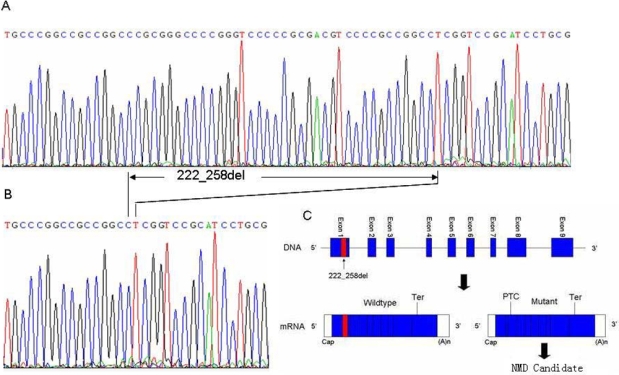
Deletion in *GPR143* identified in subject family with congenital nystagmus. **A**: Sequence for a normal family member (III: 8 in F re 1), showing the wild type allele. **B**: Sequence for the proband (III: 22), showing the 37 bp deletion from position 222 to position 258 in exon 1. **C**: The mutant transcript has a premature termination codon (PTC) located before the normal termination codon (Ter) and is likely to be degraded under the nonsense-mediated mRNA decay (NMD) mechanism.

*GPR143* was within the critical interval and was thought to be a strong candidate for mutation. Upon complete analysis of the coding and the adjacent intron regions of *GPR143,* a deletion in exon 1 was identified in all affected males (Figure 2). All obligate female carriers were heterozygous for the deletion. This deletion was not detected in normal members of the family and in other normal control subjects. It is 37 bp long and occurs at position 222 of the cDNA. This frameshift deletion introduces a subsequence premature termination codon downstream. The human *GPR143* gene encodes a protein of 404 amino acids while this deletion is predicted to result in a truncated protein with 93 amino acids. This change is predicted to result in loss of function. Our findings suggest that this *GPR143* deletion plays a causative role in the pathogenesis of X-linked CN in this family.

## Discussion

In this study, we have mapped the CN disease gene on chromosome Xp22 and identified a novel deletion in exon 1 of *GPR143*. All male patients and obligate carriers have this mutation while the other normal members of the family and the controls have not. We also screened another gene known to be involved in the development of CN, *FRMD7*, and identified no mutation. Thus, the *FRMD7* mutations should not be causative in CN development in this family. These results provide strong evidence for *GPR143* mutation in the pathogenesis of X-linked CN.

The coding sequence of *GPR143* is divided into nine exons and encodes a protein of 404 amino acids containing seven putative transmembrane domains and one potential glycosylation site using an asparagine at codon 106 [18]. *GPR143* is expressed mainly in pigment cells of the skin and eyes. It is located on the membrane of an intracellular organelle, the melanosome, found in pigment cells and plays an important role in melanosomal biogenesis [19,20]. *GPR143* mutations can cause ocular albinism, a disease characterized by severe retinal hypopigmentation, foveal hypoplasia, reduction of visual acuity, and optic misrouting [19]. This disorder is transmitted as an X-linked trait with affected males showing the complete phenotype and heterozygous females showing only minor or no retinal and cutaneous signs of the disease [19]. Thus, this disorder is described as showing an X-linked recessive pattern of inheritance in some reports [21]. Previous studies have included an extensive survey of *GPR143* mutations in a large collection of patients with ocular albinism [15,18,21,22]. The *GPR143* mutations that cause ocular albinism vary. Some are loss of function mutations, which are deletion, frameshift, and nonsense mutations [15,21,22]. Some are missense mutations that may cause defective intracellular transport or processing of the GPR143 protein [18]. However, the *GPR143* mutation in the Chinese population is rarely reported. The mutation described here is only the second *GPR143* mutation reported in the Chinese population.

Nystagmus has been reported in ocular albinism patients with mutations in *GPR143* and is thought to be a secondary phenotype in these patients [23]. However, in our family of subjects, none of the patients with the *GPR143* mutation had the classical phenotype of ocular albinism. Mutation analysis identified a 37 bp deletion from position 222 to position 258 in exon 1. This mutation leads to frameshift and introduces a subsequent premature termination codon downstream (Figure 2C). The predicted protein has only 93 amino acids and is much shorter than the normal full-length protein of 404 amino acids, suggesting that this is a loss of function mutation. Because the deletion mutation is predicted to introduce a premature stop codon, the mutant transcript is likely to be degraded by the nonsense-mediated mRNA decay (NMD) pathway [24]. If prevented by NMD, the truncated protein may not be produced. It should be noted that this hypothesis has not been tested directly as we were unable to obtain related RNA of disease tissues.

This result is consistent with that of the first *GPR143* mutation finding in a Chinese family with CN [10]. The patients in that family also had CN without the classical phenotype of ocular albinism and had a missense mutation, which resulted in the substitution of a Ser residue with a Phe residue in exon 2. This mutation occurred at a residue, which is evolutionarily highly conserved from *Xenopus* and fish to humans and was likely to cause the functional change, but the extent of its influence was unknown. As compared to that finding, the deletion found in our study, which causes frameshift and apparent loss of function, provides stronger evidence of an association between the *GPR143* mutation and CN disease in the Chinese population. In addition, the Chinese population might have a variant phenotype of mutation in *GPR143* with congenital nystagmus as the most prominent and only consistent finding. Different responses to *GPR143* mutations have been previously reported among other non-Chinese affected males (African-American and Caucasian) with different degrees of retinal hypopigmentation and other phenotypes [21]. This indicates that other factors such as genetic background and/or environmental factors may modify the phenotypes caused by *GPR143* mutations.

Our findings provide one genetic basis for CN in the Chinese population, and the *GPR143* gene should be considered in mutation testing programs for this disorder. However, the specific molecular mechanism by which these *GPR143* mutations result in CN is still unknown, and future functional studies may provide new insights.
